# Balance and its Clinical Assessment in Older Adults – A Review

**DOI:** 10.23937/2469-5858/1510003

**Published:** 2015-09-02

**Authors:** Joseph O. Nnodim, Raymond L. Yung

**Affiliations:** Department of Internal Medicine, University of Michigan Health System, USA

**Keywords:** Balance, Older Adults, Office Assessment

## Abstract

**Background:**

Human beings rely on multiple systems to maintain their balance as they perform their activities of daily living. These systems may be undermined functionally by both disease and the normal aging process. Balance impairment is associated with increased fall risk.

**Purpose:**

This paper examines the dynamic formulation of balance as activity and reviews the biological mechanisms for its control. A “minimal-technology” scheme for its clinical evaluation in the ambulatory care setting is proposed.

**Methods:**

The PubMed, Scopus and CINAHL databases were searched for relevant articles using the following terms in combination with balance: aging, impairment, control mechanisms, clinical assessment. Only articles which describe test procedures, their psychometrics and rely exclusively on equipment found in a regular physician office were reviewed.

**Results:**

Human bipedal stance and gait are inherently low in stability. Accordingly, an elaborate sensory apparatus comprising visual, vestibular and proprioceptive elements, constantly monitors the position and movement of the body in its environment and sends signals to the central nervous system. The sensory inputs are processed and motor commands are generated. In response to efferent signals, the musculoskeletal system moves the body as is necessary to maintain or regain balance. The combination of senescent decline in organ function and the higher prevalence of diseases of the balance control systems in older adults predisposes this population subset to balance impairment. Older adults with balance impairment are likely to present with “dizziness”. The history should concentrate on the first experience, with an attempt made to categorize it as a Drachman type. Since the symptomatology is often vague, several of the recommended physical tests are provocative maneuvers aimed at reproducing the patient’s complaint. Well-validated questionnaires are available for evaluating the impact of “dizziness” on various domains of patient’s lives, including their fear of falling. Aspects of a good history and physical examination not otherwise addressed to balance function, such as medications review and cognitive assessment, also yield information that contributes to a better understanding of the patient’s complaint. Ordinal scales, which are aggregates of functional performance tests, enable detailed quantitative assessments of balance activity.

**Conclusion:**

The integrity of balance function is essential for activities of daily living efficacy. Its deterioration with aging and disease places older adults at increased risk of falls and dependency. Balance can be effectively evaluated in the ambulatory care setting, using a combination of scalar questionnaires, dedicated history-taking and physical tests that do not require sophisticated instrumentation.

## Introduction

Balance impairment is common among older adults and estimates of its prevalence range between 20 and 50% [1]. In basic terms, loss of balance occurs when the center of mass (CoM) falls out of alignment with the base of support (BoS). With about two-thirds of body mass about two-thirds of body height above a relatively narrow BoS, human bipedal stance and gait are inherently low in stability. Further, the BoS is about halved when standing on one leg, adding to the potential instability. It is therefore not surprising that an elaborate biological substrate has evolved for maintaining or regaining balance.

Human beings move about as they perform their activities of daily living and are often exposed to destabilizing environmental forces. As a result, the relationship between the CoM and BoS is continually changing, thus requiring that balance be considered in a dynamic context. Rather than collapse when the line of gravity through the CoM falls outside the BoS, human beings are able to take corrective action to achieve favorable realignment. Consequently, balance is more appropriately regarded as an activity, instead of as a mere state [2].

Failure to regain balance after destabilization results in a fall. In older adults, falls are relatively common events, with 20 – 30% of them experiencing one or more falls annually. At least 10% of these events result in very serious injury such as fractures, dislocation or head injury and the mean cost of an injurious fall ranges between $3,476 and $10,749 per faller [3,4]. However, many balance-impaired older adults will come to medical attention, not because they have fallen, but because they suffer from “dizziness” - a term often used synonymously with balance impairment from a symptomatic standpoint in the medical literature [5]. In the office, the assessment of balance is often cursory and the diagnosis, unrefined, due at least in part, to time constraints. A careful reflection on the biology of balance however makes a rewarding evaluation possible, using simple tests that require only keen observation and little or no technology. Very useful information can be generated and this will greatly facilitate the task of the physical therapist who may then concentrate on the development and implementation a treatment plan. In the present article, we briefly review the basic control mechanisms of balance and outline a compact scheme for its clinical assessment by a physician in the ambulatory care setting.

## Literature Search

Three electronic bibliographic databases: PubMed, Scopus and CINAHL, were searched for balance assessment tools. The searches reached back to 2000 and included keywords and controlled vocabulary terms appropriate to each database. The first step was a high-sensitivity search of PubMed, using the following strategy: http://www.ncbi.nlm.nih.gov/pubmed?term= ((“balance tests” OR “balance test” OR “balance assessment” OR “balance assessments” OR “postural balance”[majr])) AND (older OR aged[mesh] OR elderly). It generated 5,010 articles. The searches of Scopus and CINAHL retrieved 199 and 264 articles respectively and were both overlapped by the PubMed search. We then proceeded to a refinement of the PubMed strategy to identify articles of randomized controlled trials (635). The inclusion of terms defining the clinical environment (office OR clinic) selected out 255 articles whose abstracts were reviewed to confirm eligibility.

Multiple assorted assessment tools were used in the majority of studies. Those that used equipment that are not now standard to a physician office (e.g. moving platforms, Nintendo Wii, accelerometers) exclusively (8 in number) were excluded. Also excluded were studies in which it was unclear that balance was actually measured (10 in number), the wrong “balance” (fluid, acid-base) or other irrelevant outcome was measured (4 in number). Six studies with participants under 50 years of age filtered through and were excluded. One article was in Russian and did not have an English or French abstract and another was a systematic review on exercise and osteoarthritis that did not contain enough information about its balance assessment component. Both were not considered further. In all, a total of 30 articles were excluded and the remaining 225 articles were reviewed for information about the non-instrumented office-appropriate balance tests used.

## Physiology and Pathophysiology of Balance

Balance activity is mediated by three systems: sensory, motor and central processing. The central nervous system integrates sensory inputs and generates the motor commands which control the position of the body both at station and as it moves within its environment. An impairment in any of these systems can result in a deficit in balance control. Such impairment may be due to specific pathology or the progressive decline of function in the course of normal aging.

Sensory inputs relevant to balance reach the central nervous system from the visual, vestibular and proprioceptive apparatus. Visual signals are used to create the spatial map of the environment within which objects are assessed in terms of their location, speed and direction of movement. After age 50, vision begins to deteriorate, with progressive decline in acuity, depth perception, contrast and glare sensitivity, accommodation and dark adaptation [6] Impaired depth perception is assessed to be one of the strongest risk factors for multiple falls in community-dwelling older adults [7]. As edge contrast sensitivity is lost, the propensity to trip over obstacles such as steps, curbs and cracks in the foot path increases [8]. Older adults have a significantly higher incidence of the common eye diseases - cataract (15.5%), glaucoma (3.5%) and macular degeneration (8.8%) [9].

The vestibular apparatus provides information about the position and movements of the head - the semicircular canals for angular acceleration, and the utriculosaccular system for linear acceleration as well as tilt related to gravity. The vestibulo-ocular and vestibulospinal reflexes respectively maintain visual fixation during head movements and stabilize the head during movements of the trunk and extremities. Studies of vestibular function have shown that impairment results in increased risk of falls and fall-related injuries [10]. Like vision, vestibular function deteriorates with normal aging and the prevalence of vestibulopathies increases from 49.4% in the 7th decade to 84.8% in the 9th decade [11].

Muscle spindles, Golgi tendon organs and joint capsule mechanoreceptors collect information about joint position and movement. At standstill, such proprioceptive information is considered to be the most important contributor to balance since the threshold for the perception of changes in center of pressure velocity is lower than that of the visual and vestibular systems [12]. During gait, proprioception is involved in the coordination of stepping to ensure ideal foot placement. The association between proprioceptive deficits in the lower extremities and falls is well-established [13].

Proprioceptive acuity declines with normal aging. In a recent study, older women were shown to have a 3 – 4 times higher threshold for the detection of movement at the ankle than their younger counterparts [14]. Specific pathologies which interfere with proprioception and are more prevalent in old age include peripheral neuropathy [15] and degenerative joint disease [16].

The skeletal musculature, the skeleton and its joints constitute the motor apparatus of the balance control system. Starting in the mid-twenties, there is progressive loss of lean body mass such that the cross-sectional area of the vastuslateralis for instance, decreases by approximately 40% between ages 20 and 80 [17]. Muscle strength is typically maintained at peak levels into the 5th or 6th decade. Thereafter, accelerated decline occurs, with as much as 50% lost by age 80 [18]. Lower extremity muscle weakness is highly correlated with fall risk in older adults [19] and, conversely, improvements in balance occur after lower extremity muscle strengthening exercise interventions [20].

Augmenting the BoS through stepping or grasping is one of the motor strategies for maintaining balance or recovering from its loss. If the BoS is constrained, such as when standing one-footed on a narrow beam, hip joint moments of the stance limb are used to vary the horizontal component of the ground reaction force so as to keep the CoM over the BoS [21]. Moments of force are also generated at the contralateral hip as well as the shoulders and neck to limit the angular acceleration of the head-arms-trunk complex. There is evidence that the success of these strategies is dependent on the speed with which they are implemented [22]. With advancing age however, the rate of torque development declines - by as much as 3.5% per year from age 65 [23], a process which correlates with the preferential loss of fast-twitch myofibers in aging muscle [24].

With regard to central processing, the interaction between cognition and postural control is well-established and especially manifest under dual-task conditions requiring the partitioning of attentional resources [25]. Catastrophic losses of balance are more prevalent among older adults with dementia than in their cognitively intact counterparts [26]. At a biochemical level, cholinergic mechanisms likely underlie the relationship between cognition and balance control. The cholinergic system, especially in the hippocampus and nucleus basalis of Meynert, is a specific controller of selective attention while thalamic anti cholinesterase activity plays an important role in balance control and the generation of movement [27]. Accordingly, cholinesterase inhibitors are the key drug class in the management of dementia and a few small-scale studies have shown a beneficial effect of their use in motor performance [28,29].

The sensory organs which transmit impulses to the central nervous system are prone to error due to the nature of their frames of reference. The visual system is sensitive to relative motion between the body and the environment and so may confuse environmental movement with self-motion - the illusion of vection. Likewise, the proprioceptive system, which is referenced to the support surface, is apt to generate erroneous output when that surface moves. Therefore, signaling conflict resolution is important and the central nervous system is usually able to compensate for unreliable or discordant sensory input [30].

Central processing deteriorates with advancing age. In one experiment, participants of various ages were required to walk to a target straight ahead of them under various conditions of sensory degradation (e.g. blurry vision, vestibular scrambling with transmastoidal galvanic stimulation). Whereas young adults were able to integrate the discordant inputs successfully and maintain an accurate heading to target, their older counterparts showed considerable path deviation and trunk tilt in the frontal plane [31]. The morphological correlates of central processing decline include a shrinking neuron pool and myelin loss, to the extent that the brain at age 90 is about 90% of its maximum weight [8].

## Approach to the Patient with a Balance Problem

As in every good clinical evaluation, a detailed history and thorough physical examination are the linchpin. While all components of both processes are relevant and may contribute to elucidating the problem, the present account will be confined to those elements that are addressed uniquely to balance and are often not a part of traditional history-taking and physical examination by physicians in the office. The assessment of cognition at every new patient visit, for instance, is standard practice in geriatric care. Hence, the evaluation of central processing will not be discussed further.

## History

Many older adults with balance impairment present after they have fallen but the complaint most commonly associated with defective balance is “dizziness” [5]. Unfortunately, “dizziness” is a vague term often used to describe any unpleasant sensation felt when orientation in three-dimensional space changes. It is recommended that whenever patients present with dizziness, they should be asked to describe the experience without using the terms “dizzy” or “dizziness”. Also, the emphasis should be on the very first episode because compensatory mechanisms, both adaptive and maladaptive, tend to distort the character of subsequent episodes and obscure the diagnosis.

Drachman organized the various common descriptions of dizziness into four categories (Table 1) [32].

Type 1, vertigo, is an illusion of movement. The overwhelming majority (about 90%) of the causes of vertigo are pathologies of the peripheral vestibular apparatus [8].

Type 2, presyncope, is characterized by a sensation of impending loss of consciousness due to cerebral hypoperfusion. The symptoms resolve with recumbency, except in cases of underlying cardiac disease.

Type 3, dizziness, dysequilibrium, may be sensory or motor. With the exception of cases due to visuo-vestibular mismatch, sensory dysequilibrium, unlike its motor counterpart, is exacerbated under conditions of poor ambient illumination [33].

Psychogenic dizziness (Type 4)is the least well-characterized and may well be fractional evolving forms of the other types of dizziness.^32^ In these patients, hyperventilation may induce presyncope but facial pallor is absent and the symptom persists after lying down [34].

At the time patients call to schedule their office appointments, they should be sent the scalar questionnaires shown in Table 2 which enquire about very useful clinical features of their complaint. The Activities-specific Balance Confidence (ABC) [35] scale assesses the patient’s self-rated confidence in being able to complete a panel of 16 tasks without becoming unsteady or falling. Fear of falling (so-called ptophobia) is common among older adults, including those who have never fallen [36]. It leads to self-imposed physical activity restrictions which in turn increase the risk of physical deconditioning and that of more falls. In head-to-head comparison, the ABC scale was found to be more efficient at discriminating between low- and high- mobility confidence older adults than the Falls Efficacy Scale. It has very good item responsiveness, making it very suitable for use with high-functioning community-swelling older adults [35].

The Dizziness Handicap Inventory (DHI) consists of 25 items organized into three subscales - physical, functional and emotional [37]. It thus evaluates the impact of dizziness on multiple dimensions of patients’ lives and helps to guide therapy in terms of areas of emphasis. Its high test-retest reliability makes it a good tool for assessing the effects of intervention.

## Physical Examination

Tests dedicated to the clinical assessment of balance that are appropriate to the ambulatory care setting are reviewed in this section.

### Symptom simulation

Symptom simulation is a strategy for challenging the balance control system in ways that provoke specific symptoms. This is particularly useful when the patient’s presentation is vague or poorly articulated. If, for instance, a patient admits that her symptom is reproduced when one of the simulation tests is administered, then the presentation is clarified since the mechanism of the test is known.

The head-hanging test (HHT; also called Dix-Hallpike or Nylén- Báràny maneuver) is aimed at eliciting Type 1 dizziness (vertigo). It is administered in three steps. First, the head of the seated patient is rotated to one side, facing the examiner. In this position, the plane of the ipsilateral posterior semicircular canal is oriented anteroposteriorly for maximal stimulation. Next, the patient is rapidly moved into a supine position. In the final step, the head is inclined to a position at least 10° below the horizontal and the eyes are observed for nystagmus. The test is highly specific for benign paroxysmal positional vertigo (BPPV) and the nystagmus elicited in this condition is characterized by a latency of 5 – 10 seconds, a duration of 15 – 45 seconds, a torsional or up-beating quality and extinction with repeated provocations. Some patients with otherwise classic BPPV symptoms do not exhibit nystagmus in the head-hanging position but complain of a short vertiginous spell during and after sitting up, sometimes with trunk retropulsion. This has been described as subjective or Type 2 BPPV and is considered to be due to chronic canalithiasis in the short arm of the posterior semicircular canal [38].

Upward vertical postural change is designed toprovoke Type 2 dizziness (presyncope). In orthostatic blood pressure testing, the blood pressure is measured with the supine position and after 2 minutes of standing. A drop of > 20% in mean arterial pressure has been shown to correlate better with dizziness than a > 20 torr drop in systolic or > 10 torr drop in diastolic pressure [39].

The walk-and-turn and seated head turn tests are designed to provoke Type 3 dizziness (dysequilibrium). In the walk-and-turn test, the seated patient is asked to get up, walk a distance of about 10 meters and return to her seat. If symptoms like the presenting complaint develop, then she likely has dysequilibrium. In the seated head turn test, the patient extends the neck and rotates the head slowly from side to side, as if following an aircraft flying overhead. Dizziness with this maneuver implicates the cervical spine.

### Audiologic tests

Due to the intimate anatomical proximity of the peripheral auditory and vestibular systems, audiologic testing is customarily undertaken as a component of balance assessment. In idiopathic endolymphatic hydrops for instance, a characteristic feature is combined low- and high-frequency hearing impairment which tends to fluctuate over time. A hand-held audiometer such as the Audioscope, and a 512Hz tuning fork can be used to screen for hearing loss in clinic. The Audioscope assesses hearing at the speech frequencies of 500Hz, 1000Hz, 2000Hz and 4000Hz using tones at 40dB level. Failure to hear a tone at any of the three lower frequencies (500 – 2000Hz) in either ear constitutes a positive screen [40].

The Rinné tuning fork test is recommended as an unbiased and sensitive test for both confirming audiometric findings and detecting conductive hearing loss [41]. The stem tip of the vibrating tuning fork is applied to the mastoid process and, as soon as the patient indicates he can no longer sense the vibration, the tines are placed 1 – 2 cm from the external acoustic meatus. If the patient hears the vibration, then air conduction is better than bone conduction and conductive hearing loss is ruled out.

### Visual test

The Snellen chart is used for visual acuity testing. The test is administered with the older adult standing 20 feet from the chart and wearing corrective lenses if applicable. She reads the letters from top down and her visual acuity is the Snellen fraction of the row some but not all of whose optotypes she is able to read. A visual acuity of 20/40 or less is a positive screen.

Visual acuity however, is only one of several balance-relevant visual parameters. Others (visual field, depth perception, contrast and glare sensitivity) are assessed only if the history is suggestive and referral to an ophthalmology service is usually necessary.

### Vestibular tests

Clinical vestibular tests use nystagmus and truncal sway or deviation as markers of vestibulo-ocular and vestibulo-spinal dysfunction respectively.

The head-hanging test (HHT) has already been described. The head impulse or head thrust test (HIT or HTT) is administered with the patient supine and fixated on a visual target directly above the head. The examiner than rotates the head from a position about 30º to one side, to one 30º to the opposite side. The eyes are observed for nystagmus immediately upon cessation of head movement. Normally, the amount of eye movement needed to regain fixation is very small. Vestibular derangement is present if such movement is excessive or asymmetric. The HIT has low sensitivity (54%) but is very specific (100%) [42].

Two tests are addressed to the vestibulospinal system namely, foam posturography and the Fukuda stepping. Foam posturography is an adaptation of sensory organization testing which uses the technique of sway referencing to force reliance on the vestibular apparatus for the maintenance of upright stance [43]. The individual stands on a dense foam pad about 10cm thick with arms folded across the chest and is observed for sway, first with eyes open and then with eyes closed. The compliance of the foam pad degrades somatosensory input and, with eyes closed, only vestibular signals are available for orientation. Excessive sway suggests a disorder of the vestibulospinal system. The patient must be spotted during the test, in case of catastrophic loss of balance. Foam posturography has high sensitivity (95%), specificity (90%) and correlation (p<0.005) with laboratory-based platform posturography.

In the Fukuda stepping test, the patient is instructed to march in place with eyes closed and both upper extremities extended forward. After 60 steps, any rotational movement is noted. If it is greater than 45º, vestibulospinal dysfunction is implicated [44]. Fore-aft movement is not considered abnormal.

### Functional performance tests

Function performance tests are based on postural activities and movements which occur in the course of everyday life. The timed tests measure how long a patient can maintain a given static posture, perform a given stepping sequence or walk a set distance. The Romberg test is performed in bipedal stance. In the standard test, the patient is first observed for about 30 seconds with eyes open, feet together in parallel and arms by the side. Note is made of any sign of unsteadiness such as sway or stepping. Then the observation is repeated with the patient’s eyes closed. The test is positive (i.e. Romberg sign is present) if the patient is steady with eyes open but considerably unsteady with eyes closed. The patient must be spotted during the test. Romberg sign is typically present in sensory ataxia due to proprioceptive impairment or loss of dorsal column integrity. It may also be elicited in vestibular ataxia, in which case the swaying is delayed in onset, of small amplitude and in one direction which changes with head movements. If the patient is very unsteady with eyes open, then the ataxia is probably cerebellar [5].

The unipedal stance test (UST) assesses balance in the frontal plane which is pertinent to the swing phase of bipedal gait. The patient is instructed to balance on a foot of their choosing for as long as possible. The time decreases from > 30 seconds in the third decade, to 14.2 ± 9.3 seconds in the eighth decade [45]. It is also correlated with frailty, peripheral neuropathy and risk of injurious falls [46,47]. The UST is very popular, due largely to its simplicity. However, if it is administered without standardization of the initial interpedal stance width, discrimination between groups is suboptimal [48].

The four-square step test (FSST) is used to assess dynamic standing balance. It was developed using a population of community-dwelling older adults [49] and has since been validated in other groups, most recently in persons recovering from stroke [50]. It entails stepping over four canes 2.5 cm thick laid out to form a cross, moving sequentially from one square to the next and completing two circuits in opposite directions (clockwise, then counterclockwise). The individual is required to face the same direction and must be bipedal in each square. The performance andis timed and in the original study, a cutpoint of 15 secs identified multiple fallers with a positive predictive value of 86% and a negative predictive value of 94% [49].

The timed up-and-go test measures the time taken by the seated individual to stand up from a standard armchair (seat height - 46 cm; armrest height - 65 cm), walk a distance of 3 meters quickly but safely, turn, walk back to the chair and sit down [51]. It evaluates dynamic balance and most healthy adults will complete the test in < 10 seconds. Community dwelling is considered unsafe for persons who take > 20 seconds. Using a cut point of 13.5 seconds, the test has a sensitivity and specificity of 87%, correctly identifying 13 of 15 fallers as well as 13 of 15 non-fallers.

The prototypical reaching test is the functional reach test (FRT) [52]. It measures the maximum distance the patient can reach in the forward direction with a closed fist at shoulder height beyond arm’s length without movement of the feet. It assesses dynamic standing balance and correlates well (Pearson r = 0.71) with center of pressure excursion which is the laboratory measure of the stability margin. A distance of < 6 inches is predictive of falls.

### Ordinal scales

Since balance is a complex construct requiring the interaction of multiple systems, no one test can capture all of its characteristics. It stands to reason therefore that aggregating the functional performance tests into batteries and scoring them on an ordinal scale instead of nominally, would be more informative. Two of the best-validated scales are the Performance-Oriented Mobility Assessment (POMA) and the Berg Balance Scale (BBS). Others are the Brunel Balance Assessment (BBA) and the mini-BESTest (Table 3).

The POMA [53] consists of 2 subscales: balance and gait. There are nine items on the balance subscale and all are maneuvers used in the performance of everyday activities. With the exception of three items, scoring is on a 3-point ordinal scale, with 0 indicating the highest level of impairment and 2, independence and efficacy. The exceptions are sitting balance, standing with eyes closed and turning through 360º which are scored on a binary scale. The gait subscale consists of 8 items, all but two of which are scored on a binary scale. Gait path and trunk sway are scored on a 3-point scale. Scores in both subscales are interpreted in aggregate. The POMA takes only about 10 minutes to complete. It is therefore ecologically sound and can fit into most clinic schedules.

The BBS comprises 14 items [54]. Half of them assess the ability to perform specific tasks such as transfers, reaching and picking up from the floor. The other half assess the ability to maintain positions of increasing difficulty by either decreasing the BoS or sensory input. Scoring is on a 5-point scale based on the ability to accomplish the task independently and meet some time or distance requirements. Item 1 for instance, assesses sit-to-stand transfer. The score is zero if moderate to maximal assistance is needed to stand, and 4 if the patient is able to stand without use of hands as well as stabilize independently. A score below 45 is predictive of falls.

The BBS is perhaps the best known balance assessment instrument. It has been validated in diverse groups and settings including community-dwelling older adults, institutionalized older adults and stroke patients. However, there would appear to be considerable redundancy in its item complement and rating structure. Kornetti and colleagues showed that performance in only four of the 14 items was critical for achieving a score at or near the cutoff of 45 [55]. It takes about twice as long as POMA to complete.

The BBA [56] was designed with the shortcomings of the BBS in mind. It comprises 12 balance tasks organized in a challenge hierarchy, with task difficulty progressed by BoS reduction (e.g. sitting to bipedal stance to unipedal stance) and increase in complexity (e.g. static supported to static unsupported to dynamic). It meets very strict scaling criteria but unfortunately, has only been validated in the stroke population, to be best of our knowledge.

The Mini-BESTest is a 14-item derivative of the much longer Balance Evaluation Systems Test [57]. The items are scored on a 3-point scale (0 – 2) and are focused on the assessment of dynamic balance control. Among them is a modified TUG test, sharpened for divided attention testing by the concomitant performance of serial 3’s. The estimated time investment is modest (10 – 15 mins). In comparison with the BBS, using a convenience sample of inpatients mostly with Parkinson disease and post-stroke hemiparesis, the mini-BESTest showed a lower ceiling effect and was slightly more reliable.^58o^ It was also more accurate in identifying patients who had shown significant improvement after undergoing rehabilitation. Its psychometric properties are impressive (Table 3). However, validation in other populations, especially in non-post-acute care settings, is awaited. Only 6 of the 93 patients in the study by Godi and colleagues [58] had the kind of balance problem (“unspecified age related”) which is of particular interest to geriatric care providers in the ambulatory setting.

## Clinical Balance Assessment

**Figure 1:**
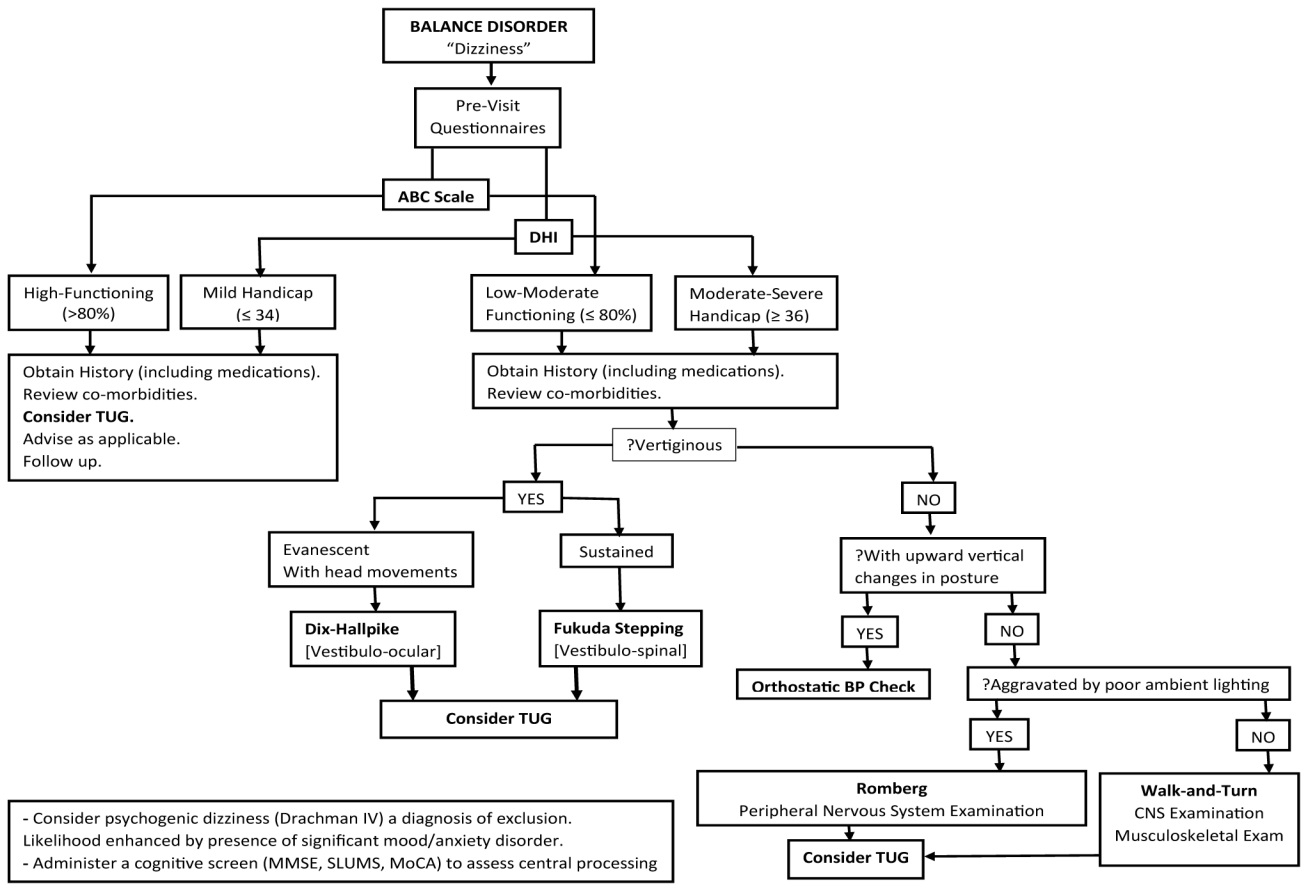
Flowchart of clinical balance assessment.

Figure 1 depicts the proposed format for balance assessment in the ambulatory care setting.

The pre-visit questionnaires (ABC scale, DHI) evaluate the impact of balance impairment on the daily life of the patient. It is important for this to be determined since arguably, the best index of symptom severity is the degree of functional limitation associated with it.

Obtaining a good history is essential, not only because it is sensible traditional clinical practice, but also because it can yield insight to the underlying pathophysiology and suggest the most informative physical examination. It is noteworthy that the presentation can sometimes be very nondescript, with the patient unable to articulate his symptom in clear terms. Such a scenario would require that provocation tests be administered in an attempt to reproduce the patient’s symptoms. The review of the patient’s medications and comorbidities is pertinent.

During the physical examination, blood pressure is measured in the supine and then upright positions to assess orthostatic tolerance. The Walk-and-Turn test seeks to provoke the symptom of the patient who might have disequilibrium and complain that she is “unsteady on her feet”. Such a patient will then benefit from close sensorimotor examination of the lower extremities. The Walk-and-Turn test is also an opportunity for gait assessment.

The Fukuda stepping test evaluates the integrity of vestibulospinal coordination. It requires no instrumentation whatsoever (“notech”) and so is preferred to foam posturography.

The Romberg test is recommended for the assessment of static balance which is impaired in the various ataxic syndromes which manifest as dysequilibrium. The unipedal stance test discriminates poorly if the initial interpedal stance width is not standardized – which usually is the case.

The TUG assesses dynamic stability and provides a global measure of functional mobility. Its administration is highly recommended as a standard component in balance evaluation. Among its superior psychometric qualities are a normal distribution, the lack of a ceiling effect and an apparent relationship to executive function [59].

## Future Directions

Body-worn inertial sensors, including linear accelerometers and gyroscopes, promise to enhance the assessment of balance in the ambulatory care setting. These devices broaden the scope of balance control parameters that can be evaluated and enable a more profound level of analysis. For instance, anticipatory postural adjustments which are the feed forward balance control mechanism that begin to accelerate the center of body mass towards the stance limb before the swing limb lifts off, can be detected and measured by accelerometry [60]. A composite test like the Timed-Up-and-Go which comprises transfers, gait and turn, but yields one metric, can be decomposed and information obtained about each of its components using the so-called iTUG protocol [61]. It is hoped that in the near future, wearable inertial sensors will become widely available, providing data at a level of precision comparable to that of three-dimensional motion analysis and force plate systems at a very small fraction of the cost. At the present time, the number of research studies in this area remains small and questions regarding type of equipment, optimal placement and the most informative outcome measures are still being addressed [62].

## Figures and Tables

**Table 1:** Classification of Dizziness

Type	Features		Etiology

1. Vertigo	Rotary or tilting sensation		Labyrinthiasis (often viral)
	Episodic		BPPV^a^ (canalithiasis, cupulolithiasis)
	Possible associations:	nystagmus	Labyrinthine ischemia (posterior circulation stroke)
		oscillopsia	Endolymphatic hydrops (e.g. Meniere disease)
	Vegetative symptoms:	nausea	Ototoxicity (if damage asymmetric)
		Vomiting	Trauma
		Pallor	Central vestibular connections (about 10% of cases)
		diaphoresis	

2. Presyncope	Sensation of impending loss of consciousness		Dehydration
	Gradual onset (except if cardiac)		Orthostasis
	Resolution with recumbency (except if cardiac)		Vasovagal phenomena
	Associations:	generalized weakness	Sympatholytic drug therapy (alpha blockade)
		visual dimming	Primary autonomic insufficiency
	Vegetative symptoms (as with vertigo)		

3. Dysequilibrium	Unsteadiness while standing or walking		Proprioceptive deficit (e.g. peripheral neuropathy)
	Exacerbated by poor lighting if sensory		Visuo-vestibular mismatch (e.g. use of optical devices)
			Compensated unilateral or balanced bilateral vestibulopathy
			Dementia
			Central motor disorders (stroke, Parkinson disease)
			Musculoskeletal disorders (e.g. DJD^b^, myopathies)
			Neuromuscular junction disorders (MG^c^, L-ES^d^)

4. Psychogenic	Vague sensation of giddiness or dissociation		Anxiety disorder
	Protracted or continuous with periodic flares Trigger often identifiable (crowds, confined spaces)		Mood disorder
	May be induced by hyperventilation		
	Associations:	anxiety (acute or chronic)	
		“light-headedness”	
		“heavy-headedness”	
		“wooziness”	

a Benign paroxysmal positional vertigo;

b Degenerative joint disease;

c myasthenia gravis;

d Lambert-Eaton syndrome

**Table 2:** Scalar Questionnaires for Balance Assessment

Instrument	Components	Scaling/Scoring		Psychometrics

Activities-specific Balance Confidence (ABC) Scale	16 items	Item:		At cutpoint of 67% (fallers vs non fallers):
		0% No Confidence		
		100% Complete Confidence		Sensitivity 84%
		Aggregate:		Specificity 87%
		< 50%	Low-functioning	
		50–80%	Moderate level	
		> 80% High-functioning		

Dizziness Handicap Inventory (DHI)	Subscales:	Item:		Internal consistency: Cronbach alpha 0.89
	- Physical 7 items	0 = No		
	- Functional 9 items	2 = Sometimes		Test-retest reliability: Pearson r > 0.80
	- Emotional 9 items	4 = Yes		
		Aggregate:		
		16 – 34	Mild Handicap	
		36 – 52	Moderate Handicap	
		≥ 54	Severe Handicap	

**Table 3:** Ordinal Scales

Battery	Components	Scoring	Psychometrics

Performance-Oriented Mobility Assessment (POMA)	Balance subscale = 9 items	Range: 0 – 28	Reliability: Pearson r =0.85
	Gait subscale = 8 items	≤ 18 - High fall risk	Validity: r = 0.91 (vs BBS)
	Takes ~10 mins to complete	≥ 25 - Low fall risk	

Mini-BESTest	Fourteen items	Item score: 0 – 2	Test-retest reliability ICC = 0.96
	Takes 10 – 15 mins to complete		Inter-rater reliability ICC = 0.98
			Convergent reliability = r = 0.85 (vs. BBS)

Berg Balance Scale (BBS)	Task performance = 7 items	Range: 0 – 56	Reliability: kappa coefficient = 0.98
	Posture maintenance= 7 items	≤ 20- High fall risk	Validity: r = 0.91 (vs POMA)
	Takes ~20 mins to complete	21 – 40- Moderate fall risk	Internal consistency: Cronbach α = 0.96
		> 40 - Low fall risk	

Brunel Balance	Hierarchy of 12 items Takes ~10 mins to complete	Pass/fail at each level Patient progresses to next level until failure	Coefficient of reproducibility^1^= 0.99 Assessment Coefficient of scalability (subjects)^2^ = 0.88 (BBA) Coefficient of scalability (items)^2^= 0.69 Item-Total Correlation = 0.34 – 0.84 Internal consistency: Cronbach α = 0.92 Reliability (kappa coefficient) = 1.0 Validity (Spearman rho; vs BBS) = 0.97

1 coefficient of reproducibility is the likelihood that the patient will fail all the items following the final item passed and pass all the items preceding it.

2 coefficient of scalability is the proportion of scaling errors to “maximum errors”. Maximum errors are extreme scores - the number of subjects who pass or fall all items or the number of items passed or failed by all subjects.

Both coefficients and the step-wise negative correlation between pass rate and task difficulty are measures of hierarchy.
